# Thiamine Allocation and Deficiency Status Throughout the Life Cycle of Cod

**DOI:** 10.1002/ece3.72828

**Published:** 2026-01-11

**Authors:** Marc M. Hauber, Vittoria Todisco, Oscar Nordahl, Petter Tibblin, Emil Fridolfsson, Elin Kärvegård, Samuel Hylander

**Affiliations:** ^1^ Centre for Ecology and Evolution in Microbial Model Systems (EEMiS) Linnaeus University Kalmar Sweden

**Keywords:** allocation, Atlantic cod, deficiency, life cycle, life history, micronutrient, thiamine, vitamin B1

## Abstract

Several wild bird and fish species across the Northern Hemisphere have been shown to episodically be thiamine deficient. This may lead to mass‐mortality events, especially in offspring. To understand the mechanisms underlying thiamine deficiency we need a better understanding of the dynamics and somatic allocation of the vitamin. Here we focus on a common, ecologically and economically important species, that is, Atlantic cod ( *Gadus morhua* ), which has been suggested to be sensitive to thiamine deficiency. We sampled cod of varying sizes and maturity stages in a system where thiamine deficiency regularly occurs (i.e., Baltic Sea) and compare these with cod from the North Atlantic, where this deficiency has not been recorded. Results show that thiamine concentrations were tissue‐specific. Concentrations in muscle and liver generally declined during growth and maturation, whereas concentrations in gonads increased. Of the total thiamine in a female's body, approximately 70% of the total pool was allocated to the gonads at the onset of reproduction, suggesting that micronutrients constitute a major investment when spawning. Free thiamine was the dominating vitamer in gonads and increased in proportion of total thiamine as gonads developed, whereas the muscle and liver's relative composition of vitamers was constant with thiamine diphosphate dominating. Transketolase activity and latency suggest that livers were saturated with thiamine and there was no evidence of ongoing thiamine deficiency. Likewise, thiamine concentrations were similar between areas with different histories of thiamine deficiency when accounting for differences in size and reproductive state, suggesting that thiamine statuses were comparable. We show that life cycle and tissue‐specific dynamics in thiamine concentrations should be considered when assessing the thiamine status of a species. Furthermore, we discuss how specific life history traits related to spawning may put species at higher risk of thiamine deficiency.

## Graphical abstract

Throughout the life cycle of fish, the allocation of micronutrients like thiamine (vitamin B1) shifts. Especially reproduction appears to be taxing, as any excess thiamine gets allocated to reproductive tissues while somatic tissues are kept at functionally sufficient concentrations.

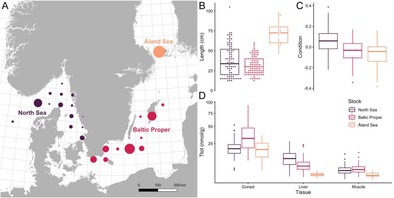

## 1 Introduction

Throughout the last centuries, an increasing number of threats to our planetary biodiversity have emerged. One of these threats is the increasing prevalence of juvenile mass mortality events in wildlife (Sutherland et al. 2018). First detected in salmonid rearing facilities in the Great Laurentian Lakes in the late 1960s, these events later appeared in the Baltic Sea, the New York Finger Lakes, and the Pacific Ocean (Bengtsson et al. 1999; Harder et al. 2018; Mantua et al. 2021). During the 1990s, these events were attributed to a lack of vitamin B1, that is, thiamine deficiency (Bylund and Lerche 1995; Fisher et al. 1995; Fitzsimons 1995; Bengtsson et al. 1999).

As with most vitamins, animals need to obtain thiamine from the environment. In an aquatic ecosystem, microbial organisms, that is, bacteria, fungi and phytoplankton, produce thiamine which then needs to be taken up by all higher trophic levels (Sañudo‐Wilhelmy et al. 2014; Fridolfsson et al. 2019; Hylander et al. 2024). This uptake has been suggested to be dominated by dietary sources but might also come via diffusion from the water or from a symbiotic gut microbiome (Kraft and Angert 2017; Hylander et al. 2024). Thiamine deficiency manifests as metabolic dysfunction, neurological symptoms or at worst mortality depending on its severity (Bengtsson et al. 1999; Balk et al. 2009; Harder et al. 2018). Throughout the last decades, a variety of organisms, including common eider ( *Somateria mollissima* ) and Atlantic cod ( *Gadus morhua* ), have been suggested to suffer from this deficiency, though most research continues to focus on salmonids such as Atlantic salmon ( *Salmo salar* ) or lake trout ( *Salvelinus namaycush* ) (Fitzsimons et al. 1999; Balk et al. 2009, 2016; Harder et al. 2018; Engelhardt et al. 2020; Todisco, Fridolfsson, et al. 2024).

Thiamine concentrations in tissues and therefore the prevalence of thiamine deficiency vary substantially between/within fish species, populations, and years (Majaneva et al. 2020; Hylander et al. 2024; Todisco, Fridolfsson, et al. 2024). Part of this variation has been attributed to external factors such as the quality and type of prey items; however, a debate on the importance of these different factors is on‐going as the mechanisms leading to a deficiency are not well described (Harder et al. 2018). In humans, it is well documented that the demand for micronutrients, like thiamine, changes throughout the life cycle and with life history events (Biesalski Hans and Jana 2018). Conversely, in wildlife, little is known about how thiamine concentrations vary naturally among life stages, that is, the ontogeny, and within an individual's different tissues. This is unfortunate given that thiamine allocation strategies and demands can be expected to vary significantly throughout the life cycle of an animal regardless of any potential deficiency. Hence, this knowledge gap hinders the determination of critical thresholds for thiamine and our understanding of when or where a deficiency might manifest.

Studies in salmonids have investigated the thiamine dynamics across part of the life cycle (Vuorinen et al. 2020; Fitzsimons et al. 2021; Todisco, Fridolfsson, et al. 2024). In doing so, Todisco, Fridolfsson, et al. (2024) introduced a comparative approach that included populations from environments where thiamine deficiency has not been observed along with populations where deficiency regularly occurs. This study design is rarely used in thiamine research and helps to differentiate whether changes in thiamine dynamics are indicative of thiamine deficiency or simply part of an organism's life cycle progression. However, there is little to no information about if and how thiamine concentrations change during the life cycle of other taxa, thus preventing the generalization of our understanding of thiamine dynamics and the development of thiamine deficiency.

Atlantic cod, hereafter referred to as cod, is one of these other species. Overfishing, the degradation of feeding/spawning grounds, and shifts in ecosystem structure have led to rapid declines in the numbers, size, and condition of most Baltic cod stocks during the latter quarter of the 20th century (Bryhn et al. 2022). Though strong regulations have been put in place, little improvements have been observed. Engelhardt et al. (2020) put forward evidence that cod in the Baltic Sea are severely thiamine deficient, suggesting the deficiency could hamper reestablishment. However, the lack of comparisons across life stages and stocks constrains firm conclusions about the prevalence and impact of thiamine deficiency in Baltic Sea stocks. Applying the previously mentioned comparative study design by Todisco, Fridolfsson, et al. (2024), we sampled cod specimens from three different systems with varying histories of thiamine deficiency in other taxa, for example, salmon. We selected specimens of varying sizes and/or maturity stages covering most of the cod's life cycle. Thiamine concentrations and vitamer ratios of muscle, liver, and gonad tissues, as well as the activity and latency of transketolase, which is used as an indicator of thiamine deficiency, were investigated. We aimed to answer how thiamine dynamics change throughout the life cycle of cod and if the investigated stocks differ in their prevalence of thiamine deficiency.

## 2 Materials & Methods

### 2.1 Study System & Sampling

**FIGURE 1:**
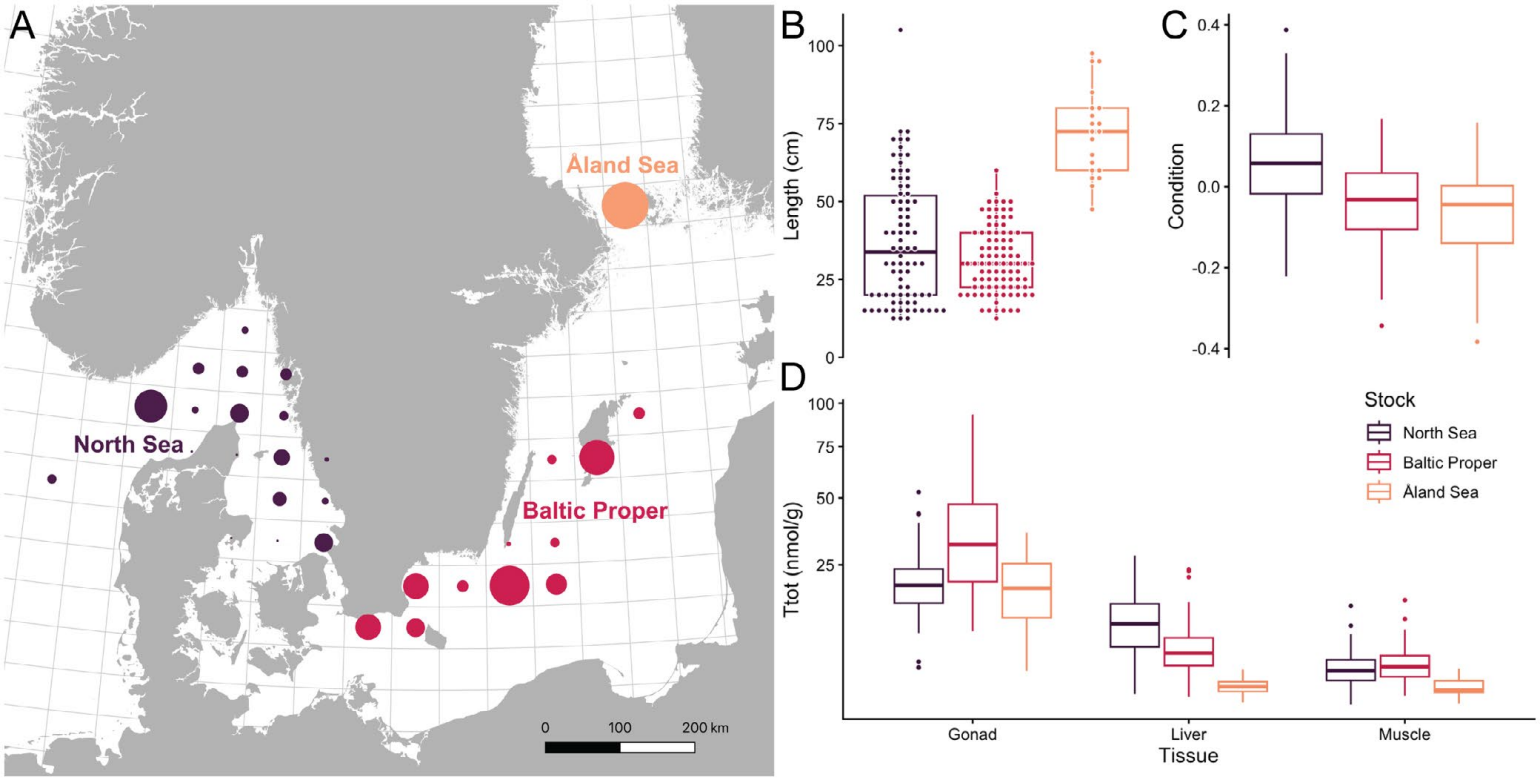
Overview of our study system and the investigated cod stocks. (A) Map of the Baltic Proper and its surrounding water bodies. Cod was sampled in ICES squares marked with a circle and separated into three different stocks: North Sea, Baltic Proper, and Åland Sea. The size of the circle represents how many fish were sampled in each ICES square, scaling from 1 to 20 specimens. (B) Length distribution of sampled specimens of the three stocks. (C) Condition of the three stocks. (D) Overview of Ttot (total thiamine concentration, nmol/g) in gonad, liver, and muscle tissue between the three stocks. Please note the y axis was square root transformed.

Cod specimens were sampled in the North Sea, Baltic Proper, and Åland Sea (Figure 1A). In the North Sea, thiamine deficiency has never been observed, and while cod stocks are declining, cod still shows good condition and grows to large sizes (Figure 1B,C). This is also true for cod in the Åland Sea, whereas most other stocks in the Baltic Sea, including the Baltic Proper, have shown rapid declines, low condition, and size truncation (Bryhn et al. 2022; Heimbrand et al. 2023). Hence, we used the North Sea and Åland Sea stocks as reference points to compare them to the Baltic Proper stock, which is suffering from aforementioned symptoms and a suggested thiamine deficiency (Engelhardt et al. 2020). Through coalescing the data on thiamine from all three stocks, we seek to better understand the extent of the potential thiamine deficiency, as well as the general thiamine dynamics throughout cod development and reproduction.

All sampled cod individuals were caught as part of environmental monitoring programs conducted by the Swedish University of Agricultural Sciences, Department of Aquatic Science (SLU Aqua). A total of 193 specimens (*n* = 193) were sampled. In the North Sea, 82 individuals were collected through bottom trawling between January and February 2022. Similarly, in the Baltic Proper, 91 individuals were caught between February and March 2022. Detailed information about these expeditions can be found in the cruise reports (Bland and Börjesson 2022; Lövgren and Casini 2022). In the Åland Sea, 20 cod were obtained through cooperation with a local fisherman using stationary deep‐water nets that were placed overnight during June 2022 (Heimbrand et al. 2023).

For each cod, the weight, length, and sex (if mature enough) were recorded. The maturity level was estimated categorically following standardized protocols (Tomkiewicz et al. 2002). For simplicity, we combined categorical maturity stages into three major maturational stages: preparation (1,2), maturation (3,4), and spawning (5–7). Liver, gonad, and gastrointestinal tract were separated and weighed to the nearest gram. Where possible, approximately 20 g of dorsal muscle, liver, and gonad tissue were vacuum‐packed and stored at −80°C for thiamine and transketolase analysis (see below). In cases where the liver or gonad weighed less than 5 g, they were weighed again on land to an accuracy of 0.001 g. Not all individuals caught during the environmental monitoring programs were sampled for this study. We selected individuals from as many size classes as possible to ensure a representative sample of the stocks' demography. To control for putative variation within stocks and/or environments, we tried to sample specimens from as many sites as possible. Individuals smaller than 14 cm were excluded from the study as their intestinal mass was insufficient for thiamine analysis.

### 2.2 Thiamine Analysis

Thiamine was analyzed in muscle, liver, and female gonad tissues according to Brown et al. (1998) with minor modifications. For a detailed description of the process see Todisco, Fridolfsson, et al. (2024). In short, tissues were homogenized and boiled in diluted trichloroacetic acid. The supernatant was washed using a mixture of ethyl acetate and hexane before adding K_3_Fe(CN)_6_ as a dye. Samples were analyzed in a Hitachi Chromaster HPLC system by measuring fluorescence. Three vitamers of thiamine were quantified: free thiamine (TF), thiamine monophosphate (TMP), and thiamine diphosphate (TDP). Concentrations (unit: nmol/g) were normalized for wet and dry weight (see below) and summed to total thiamine (Ttot).

### 2.3 Transketolase

As part of central metabolic pathways for energy production, transketolase activity levels are strongly connected to organismal functionality. Organisms produce an inactive form, that is, apoenzyme, of transketolase, which requires thiamine, more precisely TDP, as a co‐factor to be active. If an organism is thiamine deficient, some enzymes will stay inactive due to the lack of co‐factors. In this case, latency, that is, the lowered enzymatic activity due to a lack of thiamine in the system, may be observed. Hence, transketolase activity and latency are regarded as strong indicators of thiamine deficiency.

We selected 48 female cod specimens which covered the total size range and liver Ttot concentrations to measure their transketolase activity and latency. To compare the different stocks, we selected 18, 17, and 13 specimens from the North Sea, Baltic Proper, and Åland Sea, respectively. The activity and latency of transketolase were measured in liver tissues using the BCA Protein Assay Kit (ab102536, Abcam; prev. K813‐2500/5000, BioVision) and Transketolase Activity Assay Kit (ab273310, Abcam; prev. K2004‐100, BioVision). We primarily followed the manufacturer's protocols; however, we extended the protocol of the Transketolase Activity Assay Kit to also measure latency by simultaneously running replicates of each sample with and without TDP supplementation. These modifications were based on published literature measuring transketolase latency (Engelhardt et al. 2020; Jones et al. 2021).

Frozen cod liver tissue (100–200 mg) was homogenized in a 2 mL cryovial with 1 mL ice‐cold Tris buffer and 2 stainless steel balls (Steelball Lysing Matrix) for 1 min (6.5 ms^−1^; FastPrep‐24 5G, MP Biomedicals). Samples were then centrifuged at 10,000 × g at 4°C for 15 mins. The supernatant (700 μL) was collected and filtered through a 10 kDA Spin Column. Protein concentrations (μg/mL) were measured according to the BCA Protein Assay Kit. To measure the transketolase activity and latency, lysates were diluted with Tris Buffer to reach protein concentrations ranging between 0.2–0.4 μg/μL. Protein concentrations were increased from the manufacturer's recommendation (0.05–0.2 μg/μL) to reduce background noise at low activity levels. After mixing 30 μL lysate with 270 μL TKT Assay Buffer, 49 μL of the solution were placed in 5 different wells on a 96‐microtiter plate (781602, Brand GmbH & Co. KG). We added 1 μL of 10 mM TDP (freshly solubilized in TKT Assay Buffer) to two wells to measure the stimulated transketolase activity. To the other three wells, 1 μL TKT Assay Buffer was added to measure the basal transketolase activity in two wells and sample background activity in the last well. Reaction Mix (see manufacturer's protocol, 50 μL) was added to wells prepared for the measurement of transketolase activity. Background Mix (50 μL) was added to sample background controls. Standards, substrate control, and positive control were prepared following the manufacturer's protocol. We recommend adding a positive control for the latency measurement by including one sample of a specimen known to suffer from thiamine deficiency. This positive control should be prepared and run simultaneously with the actual samples. Using the microplate reader (FLUOstar Omega, BMG Labtech) preheated to 36°C, we measured the fluorescence (excitation: 544 nm; emission: 590 nm) every minute for 60 min. Following the manufacturer's protocol, we calculated an average basal and stimulated transketolase activity for each specimen. The latency was calculated by dividing the basal activity by stimulated activity, subtracting it from 1 and multiplying it by 100. If latencies were > 20% (identical to a transketolase activity coefficient of 1.25), specimens were considered at risk of deficiency (EFSA NDA 2016; Jones et al. 2021).

We would like to note that, in contrast to Gustafsson et al. (2021), we observed no changes to the basal transketolase activity when tissues were frozen and stored whole.

### 2.4 Wet and Dry Weights

In cases where enough tissue remained after subsampling for the thiamine analysis, approximately 0.75 g of frozen tissue was weighed to an accuracy of 0.001 g before and after freeze‐drying. Using the difference in weight, that is, the water content, we estimate Ttot concentrations per wet or dry weight.

### 2.5 Ethics Statement

All sampling procedures followed international and national guidelines and were approved (DNRs 255‐2012, 126‐2015, 5.8.18‐06684/2020, and 950‐2022) by the Ethical Committees on Animal Experiments, Swedish Board of Agriculture.

### 2.6 Statistical Analysis

Statistical analyzes were performed using R (version 4.5.1, R Core Team 2025). Condition, used as a proxy for health, was estimated by extracting the residuals of a fitted model between somatic weight and length (Cone 1989). Both variables were log‐transformed. Individuals who could not be sexed due to immaturity (*n* = 9) were excluded from the analyzes.

To investigate how stock as well as several phenotypic variables may affect tissue‐specific Ttot concentrations (per g wet weight), we constructed an initial linear mixed model (Gaussian distribution) including a three‐way interaction between tissue type, maturity stage and sex along with 2 two‐way interactions between tissue type and stock or length (used as a proxy for age) as well as condition as a fixed effect and the fish UID as a random effect (Appendix S1). After evaluating the three‐way interaction, separate models were fitted for each tissue type (Appendix S2). These models included length, stock, condition, sex and maturity stage as fixed effects. An interaction effect between sex and maturity stage was tested for and kept in the model if significant. The model investigating Ttot concentrations in gonad tissue lacks sex as a fixed effect as it was only measured in female fish. In all models, Ttot concentrations were log‐transformed and length was scaled. To control for non‐independence among samples, models were fitted with fish UID as a random effect using the lme4 package (Bates et al. 2015). Model fit was evaluated using diagnostic plots of residual distribution with the DHARMa package (Hartig 2024). *p*‐values were computed based on robust covariance matrix estimation, that is, Wald tests. To control for potential effects of varying water content of the samples, we reran all models using Ttot concentrations per dry weight (Appendices S1 and S2).

To further investigate how the three stocks might be differing in their thiamine status and allocation, we calculated a total thiamine amount for somatic tissues per body weight. This was performed by calculating the total amount of thiamine in the liver tissue (liver weight multiplied by liver Ttot concentration) and other somatic tissues (eviscerated weight multiplied by muscle Ttot concentration used as an estimate) for each specimen. Both were combined and divided by the somatic weight. This provided an estimate of Ttot concentration per gram somatic weight. A model identical to the tissue‐specific models (see above) was fitted.

To study thiamine allocation between tissues throughout reproduction, we calculated the total amount of thiamine in gonad (female only), liver and muscle (eviscerated weight) by multiplying tissue weight with Ttot concentration for each specimen, that is, their total thiamine pool. From there, the relative proportion of thiamine allocated to each tissue was estimated. Relative allocation of thiamine as well as tissue weight was plotted against maturity stages. The absolute allocation of thiamine was plotted against length separately for each of the three major maturity categories. Similarly, the relative proportion of the three measured vitamers TF, TMP, and TDP was estimated and plotted against maturity stages. All plots were analyzed visually.

The effect of length, stock, condition, and maturity stage on transketolase activity and latency was investigated by fitting models with aforementioned variables as fixed effects.

## 3 Results

Ttot concentrations per wet weight (nmol/g) varied between 0.5 and 15.3 in muscle, 0.6 to 28.0 in liver, and 3.0 to 93.2 in gonads (Figure 1D). Comparing statistical models using concentrations per wet weight to those per dry weight indicates only minute changes in the overall interpretation (see Appendices S1 and S2; for details on the water content of tissues, see Appendix S3). Hence, in the continued results we use Ttot concentrations per wet weight, hereafter referred to as Ttot concentrations.

### 3.1 Ttot Concentrations in Different Cod Stocks

**FIGURE 2:**
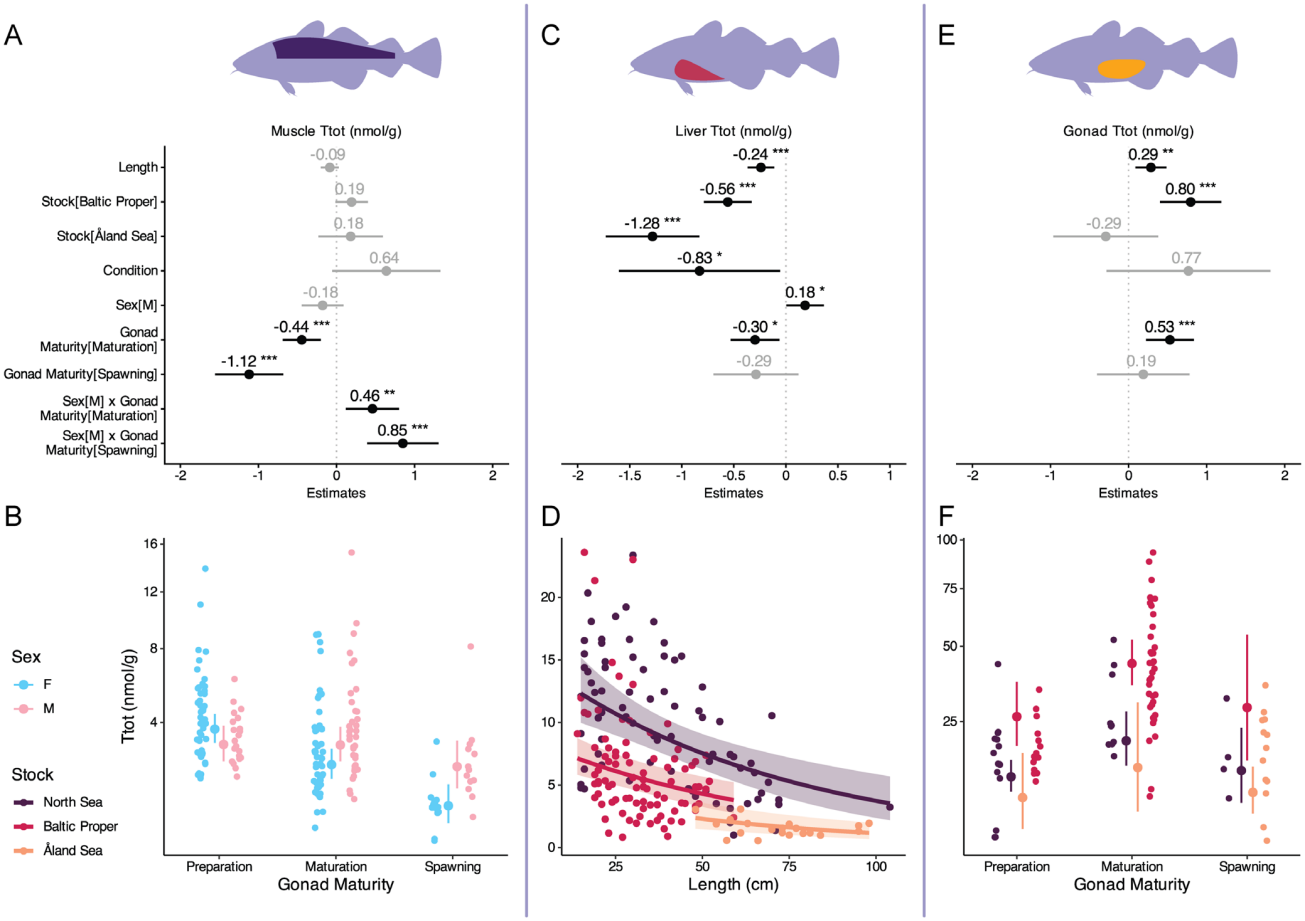
Model outputs investigating Ttot (nmol/g) in (A, B) muscle, (C, D) liver, and (E, F) gonad tissue. (A, C, E) Estimates from models in which statistically significant effects are indicated by asterisks where ***p < 0.001. Predictions of model plotted with raw data showing (B) a decline in muscle Ttot towards spawning and sex specific differences, (D) a decline in liver Ttot with size and differences between the stocks, and (F) increasing gonad Ttot during maturation and differences between the stocks. *N*‐values vary between analyzes of (A–D) somatic tissues and (E, F) gonads because Ttot concentrations of gonad tissue were measured for female fish only. (B, F) Please note that y axes were square root transformed.

Ttot concentrations differed significantly among the North Sea, Baltic Proper, and Åland Sea stocks, but differences were dependent on tissue as demonstrated by the significant interaction effect (Appendix S1). Separate analyzes per tissue showed no significant difference in Ttot concentrations in muscle across stocks (Figure 2A,B; Appendix S2). Liver Ttot concentrations were significantly higher in the North Sea compared to the Baltic Proper and Åland Sea (Figure 2C,D; Appendix S2). Contrary, gonad Ttot concentrations were significantly higher in the Baltic Proper compared to the North Sea (Figure 2E,F; Appendix S2). Gonad Ttot concentrations from Åland Sea cod were similar to the North Sea stock (Figure 2E,F; Appendix S2). Lastly, average Ttot concentrations for somatic tissues (calculation explained in section “Statistical Analysis” above) showed no significant difference between the stocks (Appendix S2).

### 3.2 Effect of Size and Condition

Throughout a cod's life cycle, we observed several tissue‐dependent relationships with size (Appendices S1 and S2). While Ttot concentrations in muscle tissue showed no significant relationship with size, liver Ttot concentrations significantly decreased with size (Figure 2A–D; Appendix S2). In gonad tissues, Ttot concentrations significantly increased with size (Figure 2E,F; Appendix S2).

The condition, a proxy for health, did not show any effect on Ttot concentrations in muscle and gonad tissues (Figure 2A,E; Appendix S2). Liver Ttot concentrations decreased with increasing condition (Figure 2C; Appendix S2).

### 3.3 Effect of Maturity and Sex

Ttot concentrations in muscle tissue decreased towards spawning but showed a significant interaction with sex (see below). In the liver, Ttot concentrations decreased significantly during the maturation phase but showed no significant difference between preparation and spawning (Figure 2C,D; Appendix S2). In contrast, gonad Ttot concentrations significantly increased during maturation (Figure 2E,F; Appendix S2). At spawning, gonad Ttot concentrations were not significantly higher compared to the preparation phase.

As gonad Ttot concentrations were only measured for female cod, the effect of sex is only investigated for muscle and liver tissues. In muscle tissue, Ttot concentrations showed a significant interaction between gonad maturity and sex (Figure 2A,B; Appendix S2). Whereas male muscle Ttot concentrations stayed constant during preparation and maturation, only decreasing at spawning, Ttot concentrations in female cod consistently decreased through maturation and spawning. This led to muscle Ttot concentrations being lower in female cod than male cod during maturation and spawning. Males had significantly higher liver Ttot concentrations compared to females (Figure 2C; Appendix S2).

### 3.4 Tissue‐Specific Thiamine Allocation Throughout the Maturity Stages

We calculated the total thiamine pool within each female (calculation explained in section “Statistical analysis” above) and estimated the relative proportion of thiamine in each of the studied tissues to understand how reproduction affects allocation of thiamine.

**FIGURE 3:**
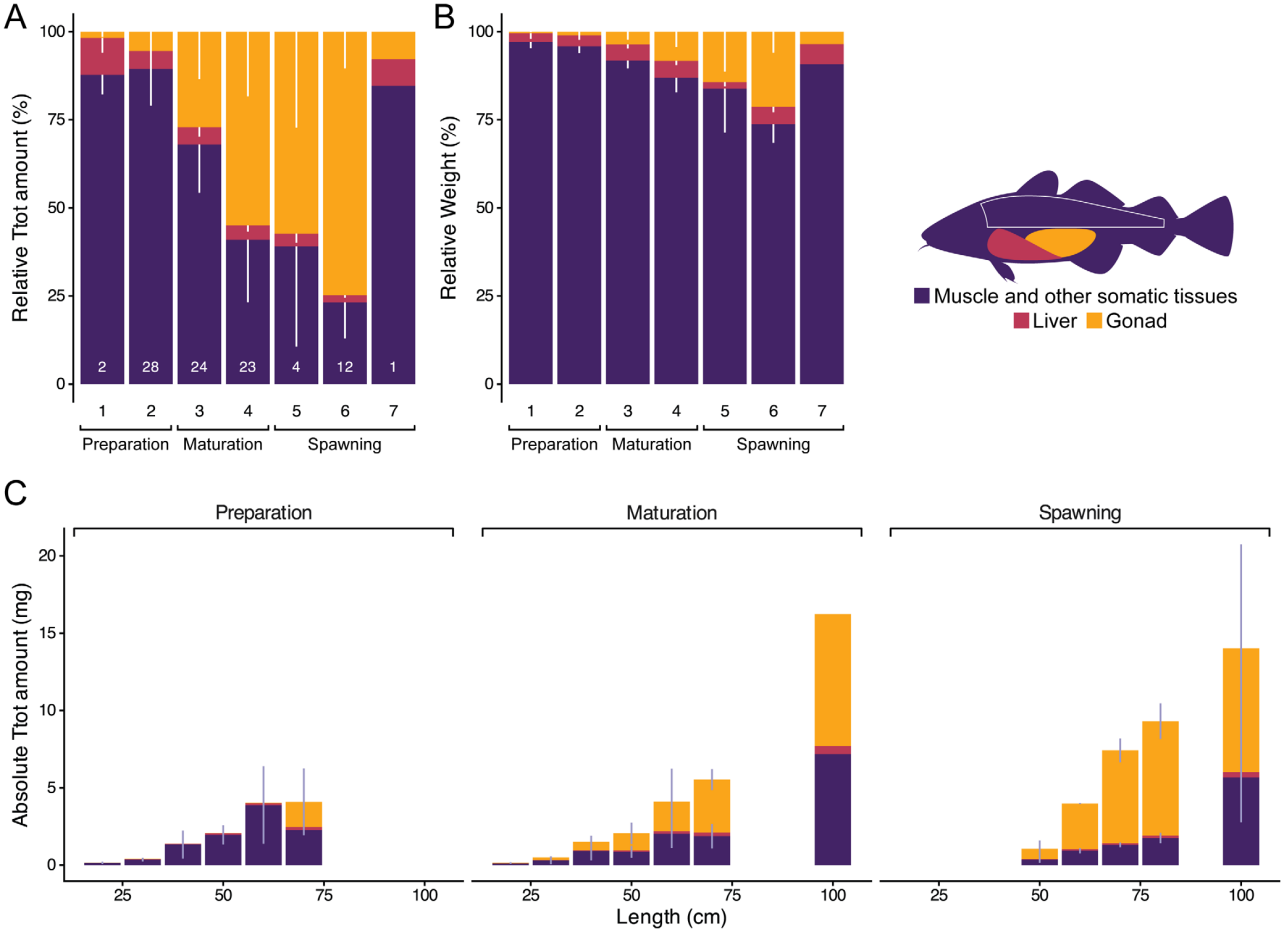
Relative and absolute allocation of Ttot to the different tissues and the relative weight of the different tissues throughout the reproduction of female cod. (A) Relative amount of Ttot allocated to the different tissues showing a steady increase in the proportion allocated to gonad tissues of up to 75%. *N*‐values are given in white at the bottom of each bar. (B) Relative weight of different tissues showing a steady increase in the gonadosomatic index of up to 25%. (C) Absolute Ttot amount allocated to the different tissues separated by 10 cm size classes and reproductive categories. (A, B) White or (C) light purple lines represent standard deviation (SD).

Females that are immature or in the preparation stage allocated most thiamine to the muscle tissues (89% ± 10%; percent ± SD) with liver and gonad tissues only containing 5% ± 3% and 5% ± 10% of the total thiamine pool, respectively (Figure 3A). Entering maturation, females increased their thiamine input to gonad tissues (41% ± 21%). While liver tissues continued to maintain 5% ± 2%, the proportion of the total thiamine pool in muscle and other somatic tissues (55% ± 21%) declined (Figure 3A). Reaching spawning, muscle and other somatic tissues as well as liver tissues showed further declines in their proportions of the total thiamine pool containing only 31% ± 22% and 3% ± 2%, respectively. At spawning, 67% ± 22% of thiamine within a female individual was allocated to the gonads (Figure 3A).

The relative proportion of the gonad weight, that is, gonadosomatic index, similarly increased from preparation to maturation to spawning but at lower proportion from 1% ± 1% to 6% ± 4% to 19% ± 9%, respectively (Figure 3B). While the relative proportion of the liver weight, that is, hepatosomatic index, stayed around 4% ± 1%, the relative proportion of muscle and other somatic tissues decreased with increasing gonadosomatic index (Figure 3B).

The absolute amount of Ttot strongly increased with length (Figure 3C). In contrast, the absolute Ttot amount in fish of the same length class but different reproductive stage did not show clear differences and appeared rather consistent (Figure 3C).

As the total thiamine pool consists of the three vitamers TDP, TMP, and TF, which carry different functions (see discussion), changes in their relative proportion may further our interpretation of the data presented above.

**FIGURE 4:**
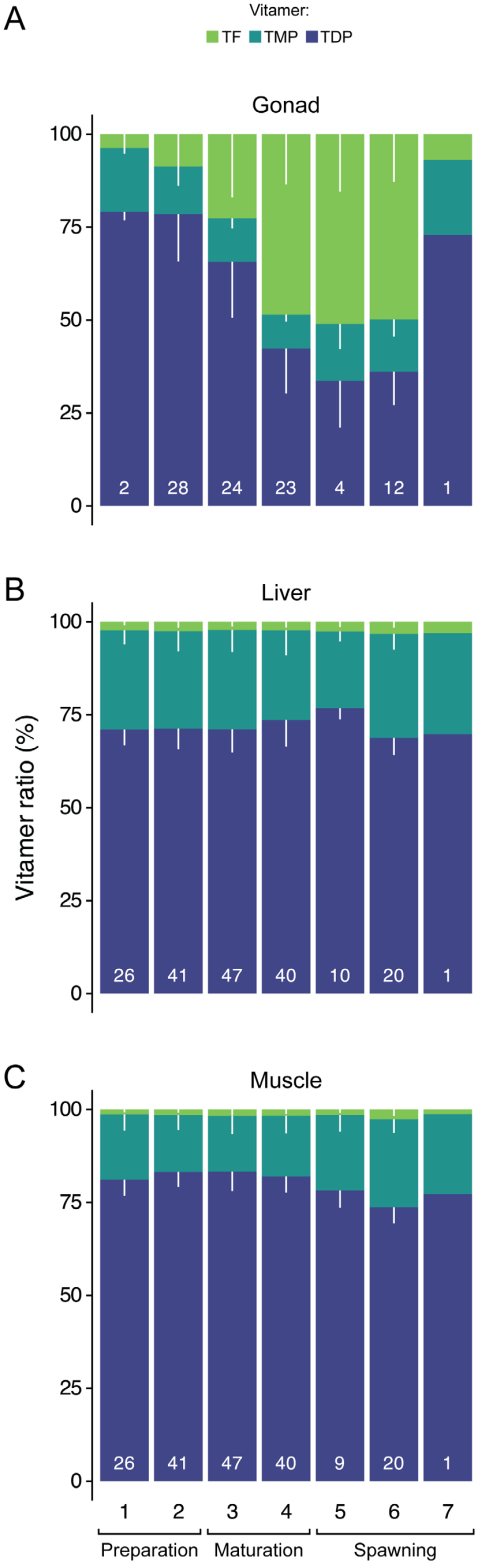
Vitamer ratios of TF, TMP, and TDP throughout the reproduction of cod in (A) gonad, (B) liver, and (C) muscle tissue. Stage of reproduction was categorized from 1 to 7 which were combined into preparation, maturation, and spawning stages for simplicity. *N*‐values are given in white at the bottom of each bar and vary between (A) gonad and (B, C) somatic tissues because thiamine concentrations of gonad tissue were measured in female fish only. Standard deviation (SD) is presented by the white lines.

In gonad tissue, the proportion of TF showed a clear increase from preparation (8% ± 14%) to maturation (35% ± 20%) and spawning (48% ± 16%; Figure 4A). While the proportion of TMP in gonad tissue stayed consistent around 12% ± 4%, TDP decreased from preparation (79% ± 12%) to maturation (54% ± 18%) and spawning (38% ± 13%; Figure 4A). In muscle and liver tissue, vitamer ratios did not show notable changes throughout the maturity stages (Figure 4B,C) and did not differ between the sexes (Appendix S4). In muscle tissue, proportions between TDP/TMP/TF stayed around 81%/16%/2% with TDP being the prominent vitamer (Figure 4C). Similarly, in liver tissue, vitamer proportions stayed around 72%/26%/2%. In liver tissues, the proportion of TMP was around 10% higher than in muscle tissues (Figure 4B).

### 3.5 Thiamine Deficiency Prevalence

As Ttot concentrations were of overall similar ranges between the different cod stocks (see above), the activity and latency of transketolase can help us better understand if and how the stocks differ in their thiamine status.

**FIGURE 5:**
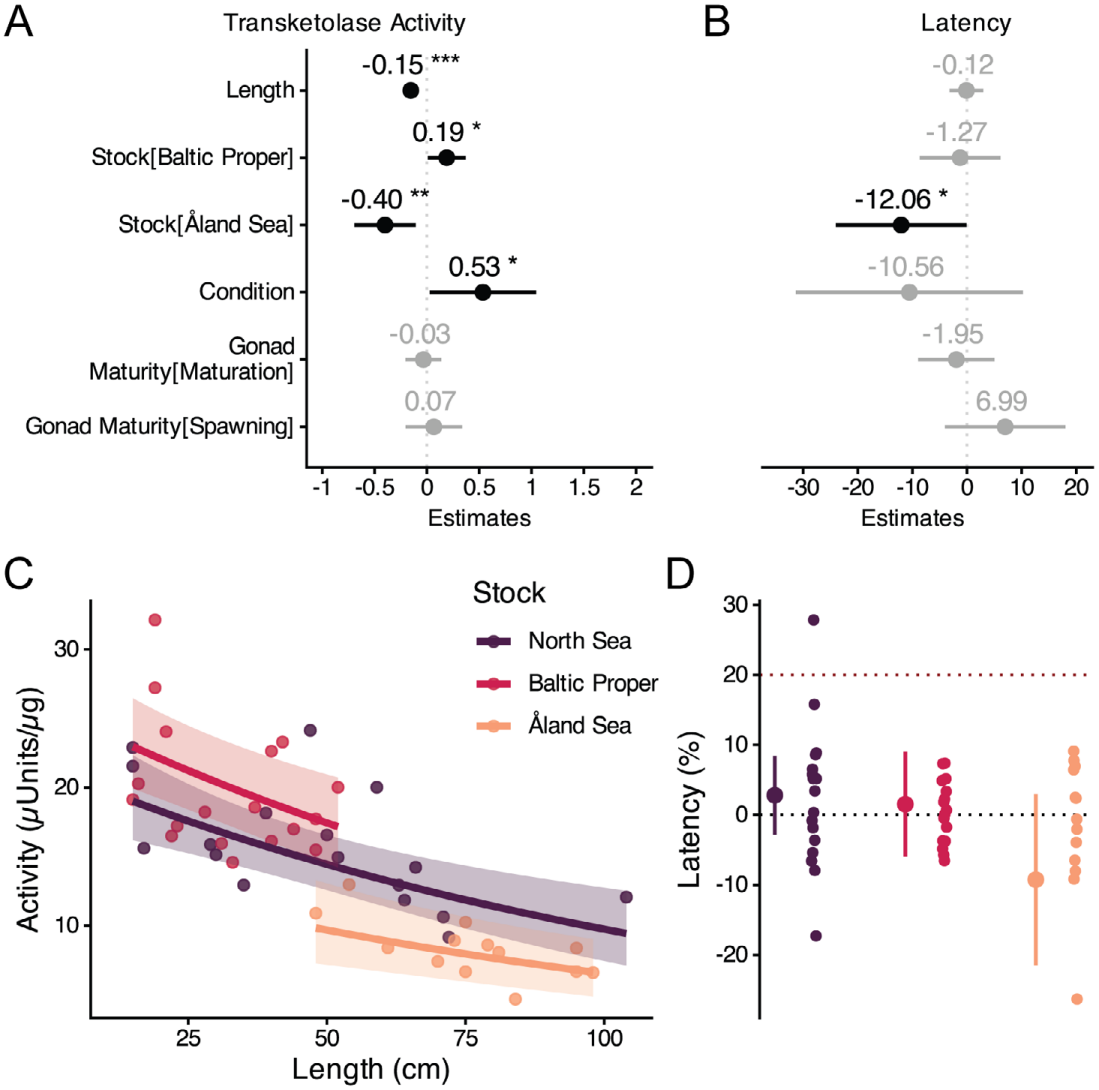
Model outputs investigating transketolase activity and latency. (A, B) Estimates from models investigating transketolase activity (A) or latency (B) in which statistically significant effects are indicated by asterisks where ***p < 0.001. (C) Predictions of model plotted with raw data showing a decrease in transketolase activity with size and differences between the stocks. (D) Predictions of model plotted with raw data showing transketolase latencies averaging around zero. Threshold indicating thiamine deficiency is represented as a red dotted line. Negative latency values are illogical but are presented to indicate range of measurement inaccuracy.

Whereas cod in the Åland Sea showed a significantly lower transketolase activity in liver tissues (Figure 5A,C), cod from the Baltic Proper had significantly higher transketolase activity compared to the intermediate North Sea stocks (Figure 5A,C). It should be noted that transketolase activity in liver tissue overall decreased with size (Figure 5A,C). Hence, the differences in transketolase activity between stocks align with their different size distribution. Transketolase activity increased significantly with condition (Figure 5A).

For almost all individuals, the transketolase latency stayed below 20% (Figure 5D). One individual in the North Sea with a latency of 28% exceeded this threshold and could be considered at risk of deficiency. Transketolase latency was significantly lower in cod from the Åland Sea (Figure 5B). This was most likely driven by the higher prevalence of negative latency values, which represent measurement inaccuracy. Overall, transketolase latency did not differ between the cod stocks and averaged around 0%.

## 4 Discussion

We investigated if and how Ttot concentrations in different tissues of cod change throughout their life cycle. Secondly, we compared Ttot concentrations among three cod stocks with differing histories of reported thiamine deficiency. Additionally, we measured the activity and latency of transketolase to understand the prevalence of thiamine deficiency in these stocks. We found that (i) Ttot concentrations of all tissues are heavily affected by the reproductive state (Figures 2, 3, 4); (ii) particularly liver Ttot concentrations and transketolase activity decreased with size/age (Figures 2D and 5B); (iii) the investigated cod stocks showed overall comparable Ttot concentrations (Figure 2) and no transketolase latency (Figure 5D), indicating that none of the stocks were suffering from thiamine deficiency.

### 4.1 Reproduction & Its Associated Life History Shape Thiamine Dynamics

Thiamine is present in different forms, so‐called vitamers, for example, TF is the vitamer associated with transport whereas TDP is the active cofactor (Manzetti et al. 2014; Kraft and Angert 2017). There was a variable vitamer mixture in gonads with a dominance of TF during spawning, whereas TDP was steadily the dominating vitamer in liver and muscle tissues. Earlier studies have mainly focused on salmonids and especially samplings during the spawning season, largely omitting the dynamics during the adult life stage of the fish (Brown et al. 1998, 2005; Amcoff et al. 1999; Fitzsimons et al. 1999; Harder et al. 2018). At spawning, female fish generally have high concentrations of TF in eggs whereas the relative mixture of vitamers in liver and muscle is dominated by TDP (Brown et al. 1998, 2005; Amcoff et al. 1999; Harder et al. 2018). As shown here, Ttot concentrations and the proportion of TF increased only in gonadal tissues towards spawning suggesting that reproduction in fish is a highly demanding process on micronutrients like thiamine. We show that allocation of thiamine to the gonads in cod is a gradual process during preparation and maturation before spawning. Overall, this suggests that micronutrient investment in gonads is significant with approximately 70% of all thiamine in the body located in the gonads at the onset of reproduction.

Simultaneously to the investment in gonads, Ttot concentrations in muscle and liver tissue decreased during maturation/towards spawning. This gradual decline most likely is driven by an increased allocation of available thiamine to the gonad tissue (Ejsmond et al. 2025). The thiamine allocated to the gonads could be sourced externally, that is, through the diet, or internally from body stores of thiamine in somatic tissues like muscle and liver (Koski et al. 2005). To our knowledge, little is known about the potential to reallocate thiamine between tissues in fish. Thiamine is mostly considered to be taken up and transported as TF and converted to TMP or TDP once it enters a cell (Reggiani et al. 1984; Rindi et al. 1984; Weber et al. 1990). Since TDP cannot permeate biological membranes due to its charge, its allocation is often considered to be final. However, the process of removing phosphate groups, that is, dephosphorylation, from thiamine has been described in a variety of organisms (Rindi et al. 1984; Schweingruber et al. 1991; Bettendorff et al. 1996; Rapala‐Kozik et al. 2009). This process appears to be especially important in tissues with shifting metabolic demands such as the nervous system (Rindi et al. 1987; Rindi et al. 1991; Nauti et al. 1997). With phosphatases converting TDP to TMP or TF, thiamine would be able to permeate cell membranes again, enter the blood stream and could be reallocated to other tissues depending on the organism's changing needs, for example, during reproduction. Within this mechanism, TDP or TMP act not only as a cofactor or metabolite; these vitamers would function as a storage that could be reallocated using dephosphorylation (Weber et al. 1990). In cod, as mentioned above, we observe a decline in muscle and liver Ttot concentrations during maturation/towards spawning. The vitamer ratios which are predominantly TDP and TMP in both tissues show no changes throughout reproduction. These observations may suggest the presence of the reallocation of thiamine between tissues. However, as cod may only reduce its feeding and dietary thiamine intake during the spawning period (Fordham and Trippel 1999; Michalsen et al. 2008), it is impossible to disentangle whether it is a reallocation of internal thiamine sources or simply an increased allocation of externally sourced thiamine (e.g., from diet) to the gonads. This increased allocation of externally sourced thiamine to the gonads may similarly lead to gradual declines in Ttot concentrations of somatic tissues such as muscle and liver due to an imbalance between degradation and supply.

Studies of fish species with long pre‐spawning fasting periods, such as salmonids, might help disentangle this mechanism. Externally sourced thiamine, at least from dietary items, can be excluded while gonads and eggs develop and the fish prepares for spawning (Kallio‐Nyberg and Ikonen 1992; Vuorinen et al. 2014). It is not known how salmonids allocate thiamine to the eggs during this period. Pharmacokinetic studies and hatchery practices of injecting thiamine or letting fish swim in thiamine bath show that thiamine entering the bloodstream indeed can reach gonads and eggs (Koski et al. 2005). Especially muscle tissue, simply due to its large proportional mass, has been hypothesized a potential source for thiamine in maturing salmonids (Koski et al. 2005). Interestingly, when investigated, muscle Ttot concentrations in Atlantic salmon did not decrease throughout the pre‐spawning fasting period (Vuorinen et al. 2020; Todisco, Fridolfsson, et al. 2024; Todisco, Hauber, et al. 2024). However, liver Ttot concentrations showed a 62% decrease in Ttot concentration in the freshwater phase before spawning (Vuorinen et al. 2020). Furthermore, the vitamer ratio in the liver tissue was predominated by TDP and TMP. These patterns suggest the potential in salmonids to reallocate thiamine between tissues via dephosphorylation. More studies are needed to test the validity of this hypothesis since other processes may also lead to the decline in Ttot concentrations or other processes could provide externally sourced thiamine. Furthermore, gonad Ttot concentrations have also been shown to drop before spawning in salmonids, which may appear counterintuitive to the allocation mechanism discussed here (Vuorinen et al. 2020; Todisco, Fridolfsson, et al. 2024). As gonads develop and grow, their Ttot concentrations can decline even though their Ttot amount increases. This is due to the increase in mass of the gonad tissue. Hence, future studies investigating this potential mechanism should consider the Ttot amount in tissues throughout the reproduction of salmonids and other species.

Overall, we suggest that species like cod that are batch spawners without a period of pre‐spawning fasting should be less likely to be constrained by thiamine compared to other species, such as salmonids, that have a long pre‐spawning fasting. During their gonad development, dietary sources of thiamine are negligible, and the fish may be limited to only their internal thiamine storage in for example, liver tissue, or as speculated other sources such as thiamine dissolved in the water and taken up via the gills, or from gut microbiota (Harder et al. 2018; Fitzsimons et al. 2021; Hylander et al. 2024).

### 4.2 How Size Affects Thiamine Dynamics

At a food web scale, it has been shown that there is a trophic dilution so that Ttot concentrations are higher in lower trophic levels (e.g., in phytoplankton) compared to higher trophic levels (e.g., in fish; Ejsmond et al. 2019; Hylander et al. 2024). Interestingly, this pattern might also apply to individuals of the same species. As an organism grows, its metabolic rate does not increase proportionally but at a slower rate, that is, allometrically (Koziowski and Weiner 1997; White and Marshall 2023). As thiamine functions primarily as a cofactor for several enzymes within the basal metabolism, we can assume its demand to scale with the aerobic metabolism and therefore size of an organism (Patel 2019). In our study, this was especially apparent in liver tissue, where Ttot concentrations and the activity of the thiamine‐dependent enzyme transketolase decreased with size. Engelhardt et al. (2020) observed a similar decrease of liver Ttot concentrations with age attributing it to thiamine deficiency. Likewise, several other studies have shown that Ttot concentrations decline with the size of the fish (Fitzsimons et al. 2013, 2021). These studies argue that this is due to long‐term starvation during migrations or due to long‐term exposure to thiaminase I reducing the overall thiamine status of the fish (Fitzsimons et al. 2013, 2021). We argue that a decrease in Ttot concentrations of somatic tissues with size/age of an organism is not necessarily an indicator for thiamine deficiency. As we observe this pattern in all our studied cod stocks and knowing that metabolic rate scales allometrically with size, we suggest a decrease in Ttot concentrations of somatic tissues with size to be a naturally occurring pattern. Especially since our transketolase latency estimate suggests that fish were saturated with thiamine and not under deficiency. Hence, future comparative studies investigating thiamine deficiency should control for this relationship as populations might differ in their demography.

### 4.3 The Prevalence of Thiamine Deficiency in Cod

To our knowledge, thiamine deficiency in cod has previously only been investigated by Amcoff et al. (1999) and Engelhardt et al. (2020). With Amcoff et al. (1999) investigating Ttot concentrations in liver and gonad tissues of spawning cod in the Baltic Proper (ICES SD 25, 26, and 28), few conclusions about their thiamine status could be made as reference values were lacking. Engelhardt et al. (2020) investigated Ttot concentrations in liver and transketolase activity/latency in liver as well as in brain tissues of cod in a subsection of the Baltic Proper (ICES SD 25). The reproductive stage was not categorized in that study, but as specimens were sampled in autumn, we expect regeneration to preparation stages. Engelhardt et al. (2020) put forward evidence that cod suffer from thiamine deficiency. This was based most prominently on measures of high transketolase latencies in liver (mean 15%; max 49%) and brain tissues (mean 27%; max 66%).

We observed tissue‐specific Ttot concentrations to partly differ between the North Sea, Baltic Proper, and Åland Sea. However, on the Ttot level of the organism, these concentrations even out and indicate no overall differences in thiamine levels between the stocks. Such differences have been shown in Atlantic salmon when comparing fish from the Baltic Sea with Atlantic Ocean and landlocked populations (Todisco, Fridolfsson, et al. 2024), indicating a limitation of thiamine in Baltic salmon. Transketolase enzymes in cod were saturated with TDP, that is, measured latencies were below 20%, in the investigated cod stocks. It is important to note that Engelhardt et al. (2020) considered individuals with a latency > 6% thiamine deficient. We followed a more conservative threshold of > 20% (EFSA NDA 2016; Jones et al. 2021), since it is commonly used in the medical field and because we observed method‐induced variation leading to negative latency which has no biological meaning (Figure 5D).

Overall, in contrast to Engelhardt et al. (2020), we find no evidence for thiamine deficiency in the assessed cod stocks. As it could be hypothesized that these differences between studies are due to yearly fluctuation in thiamine deficiency in the Baltic Sea (Majaneva et al. 2020) or the differing reproductive stages, we encourage further longitudinal studies on thiamine dynamics in cod including later reproductive stages, that is, regeneration. Such studies would fill important knowledge gaps of the micronutrient dynamics and potential deficiencies throughout the life cycle of cod and other fish with similar life history traits.

## Author Contributions

**Marc M. Hauber:** conceptualization (equal), data curation (lead), formal analysis (lead), investigation (lead), methodology (lead), project administration (equal), resources (lead), software (equal), validation (equal), visualization (lead), writing – original draft (lead), writing – review and editing (equal). **Vittoria Todisco:** conceptualization (supporting), data curation (supporting), investigation (supporting), writing – review and editing (equal). **Oscar Nordahl:** conceptualization (equal), formal analysis (equal), project administration (supporting), supervision (equal), validation (supporting), writing – review and editing (equal). **Petter Tibblin:** conceptualization (equal), supervision (equal), writing – review and editing (equal). **Emil Fridolfsson:** data curation (supporting), investigation (supporting), methodology (supporting), writing – review and editing (equal). **Elin Kärvegård:** investigation (supporting), writing – review and editing (equal). **Samuel Hylander:** conceptualization (equal), funding acquisition (lead), project administration (supporting), resources (equal), supervision (lead), writing – original draft (supporting), writing – review and editing (equal).

## Funding

This work was supported by Svenska Forskningsrådet Formas (Grant FR‐2020/0008), Vetenskapsrådet (Grant 2019‐04251), and Strategic Research Program EcoChange.

## Conflicts of Interest

The authors declare no conflicts of interest.

## Supporting information


**Data S1:** ece372828‐sup‐0001‐supinfo.docx.

## Data Availability

Data supporting this study are openly available from Dryad at https://doi.org/10.5061/dryad.cjsxksnjc.
