# Supplementary material for: Thiamine Allocation and Deficiency Status Throughout the Life Cycle of Cod
Source: Ecol Evol. 2026 Jan 11;16(1):e72828. doi: 10.1002/ece3.72828 (PMC12793782; doi:10.1002/ece3.72828)
Supplement: Supplementary file 1 — Data S1: ece372828‐sup‐0001‐supinfo.docx. [file ECE3-16-e72828-s001.docx]

Supplementary Material

Appendix 1: Summary table containing model output when investigating Ttot of several tissues together both normalized as wet and dry weight.


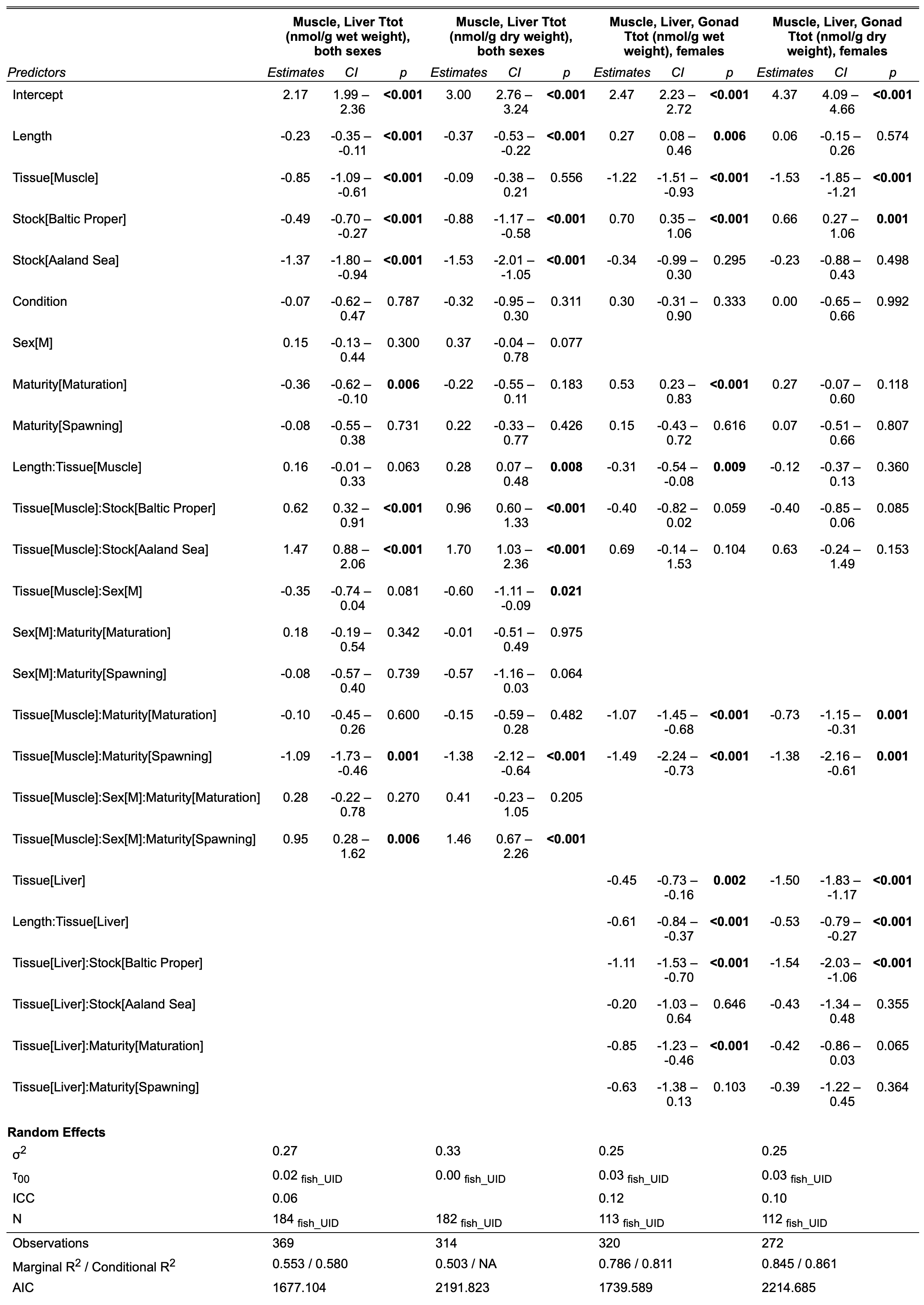


Appendix 2: Summary table containing model output when investigating Ttot of each tissue separately both normalized as wet and dry weight. Also contains model output of investigating somatic Ttot.


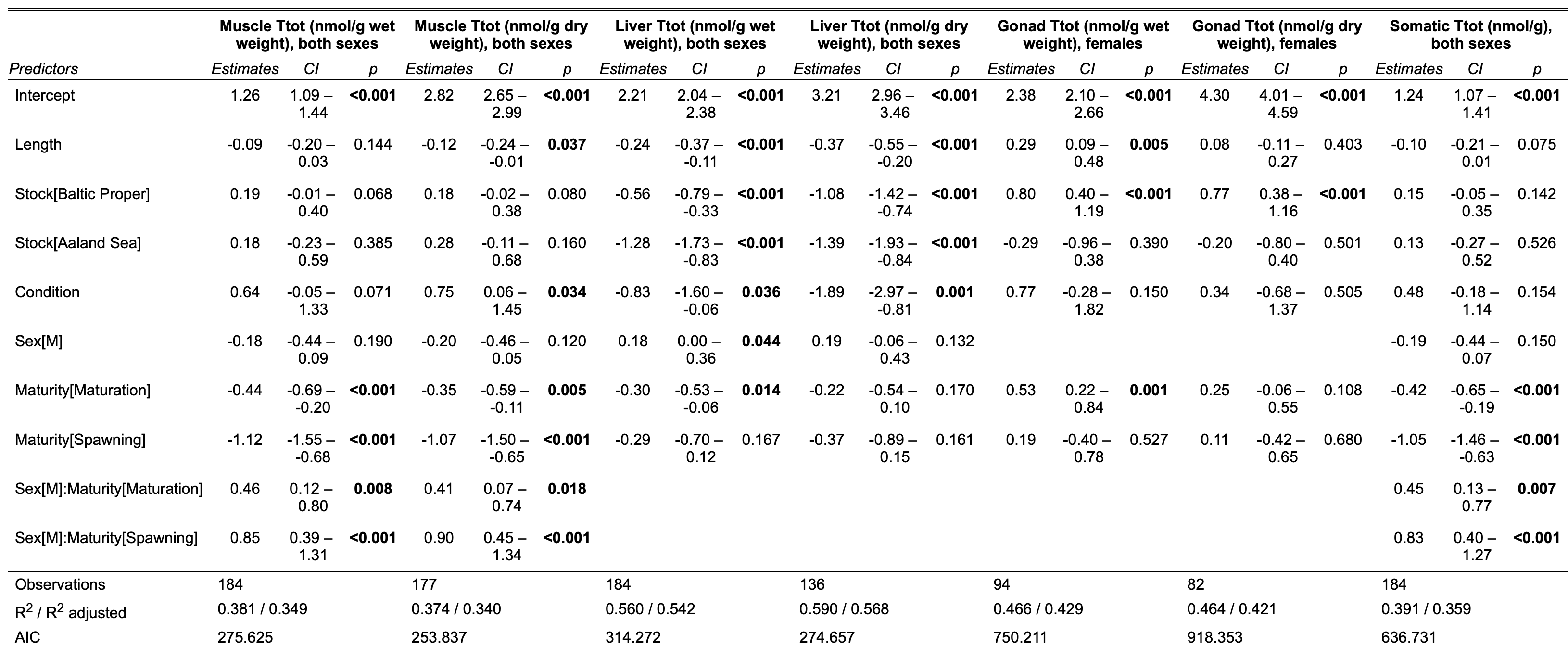


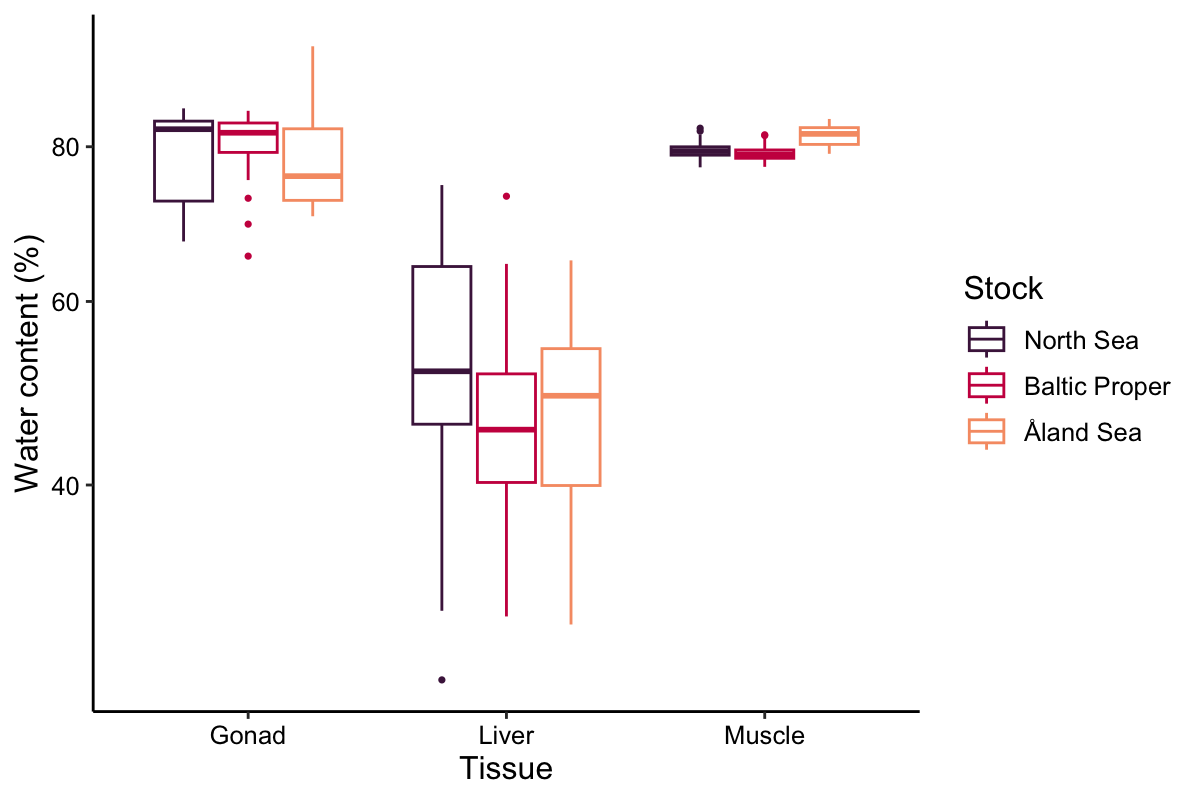


Appendix 3: Relative water content (%) of gonad, liver, and muscle tissue separated by investigated cod stock. Mean values are 80 ± 5% (percent ± SD) in gonad, 50 ± 12% in liver, and 80 ± 1% in muscle tissue.


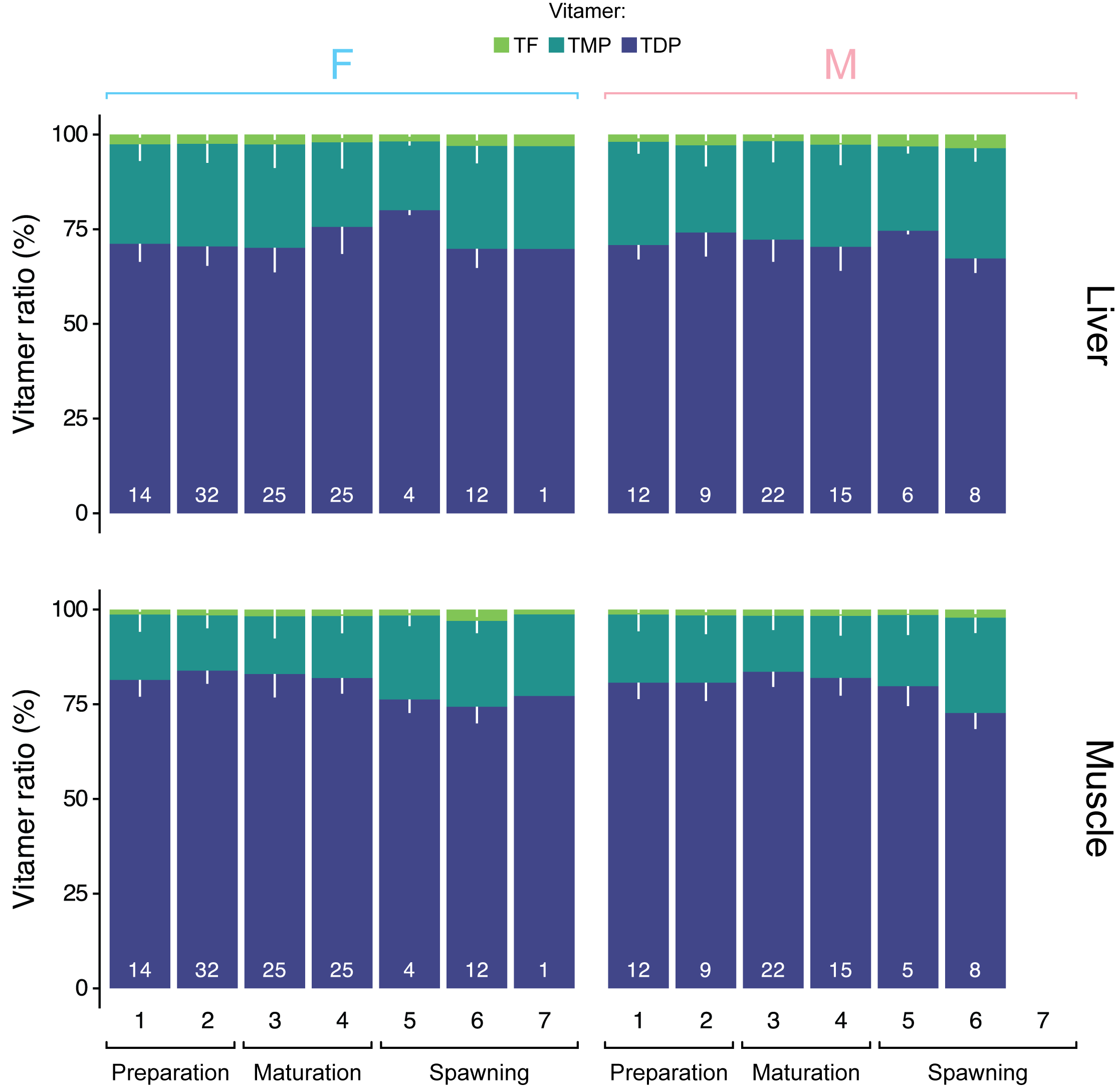


Appendix 4: Vitamer ratios in liver and muscle tissue throughout the reproduction of cod separated for female and male specimens.
